# BIX01294 inhibits EGFR signaling in EGFR-mutant lung adenocarcinoma cells through a BCKDHA-mediated reduction in the EGFR level

**DOI:** 10.1038/s12276-021-00715-7

**Published:** 2021-12-07

**Authors:** Ji Hye Kim, Jongwook Kim, Se Seul Im, Ji Hyeon Lee, Sein Hwang, Eun-Ju Chang, Dong-Myung Shin, Jin Kyung Rho, Jaekyoung Son

**Affiliations:** 1 Department of Biomedical Sciences, Asan Medical Center, AMIST, University of Ulsan College of Medicine, Seoul, 05505 South Korea; 2 Department of Convergence Medicine, Asan Medical Center, University of Ulsan College of Medicine, Seoul, 05505 South Korea

**Keywords:** Cancer metabolism, Non-small-cell lung cancer

## Abstract

BIX01294 (BIX), an inhibitor of the G9a histone methyltransferase, has been reported to have antitumor activity against a variety of cancers. However, the molecular mechanisms underlying its anticancer effects, particularly those against lung cancer, remain unclear. Here, we report that BIX induces apoptotic cell death in EGFR-mutant non-small cell lung cancer (NSCLC) cells but not in their wild-type counterparts. Treatment with BIX resulted in a significant reduction in the EGFR level and inhibition of EGFR signaling only in EGFR-mutant NSCLC cells, leading to apoptosis. BIX also inhibited mitochondrial metabolic function and decreased the cellular energy levels that are critical for maintaining the EGFR level. Furthermore, BIX transcriptionally downregulated the transcription of branched-chain α-keto acid dehydrogenase (BCKDHA), which is essential for fueling the tricarboxylic acid (TCA) cycle. Interestingly, this BCKDHA downregulation was due to inhibition of Jumanji-domain histone demethylases but not the G9a histone methyltransferase. We observed that KDM3A, a Jumonji histone demethylase, epigenetically regulates BCKDHA expression by binding to the BCKDHA gene promoter. BIX exposure also led to a significant decrease in the EGFR level, causing apoptosis in EGFR-TKI (tyrosine kinase inhibitor)-resistant cell lines, which are dependent on EGFR signaling for survival. Taken together, our current data suggest that BIX triggers apoptosis only in EGFR-mutant NSCLC cells via inhibition of BCKDHA-mediated mitochondrial metabolic function.

## Lung cancer: Overcoming drug resistance

A drug known as BIX that is effective against bladder and breast cancers may also be effective in fighting non-small cell lung cancer (NSCLC). Although advances have been made in treatment of NSCLC, one of the most effective drugs targets a protein called EGFR, and *EGFR* gene mutations that lead to acquired drug resistance are common. Jaekyoung Son at the University of Ulsan, Seoul, South Korea, and colleagues investigated whether BIX is effective against NSCLC and attempted to elucidate its mechanism of action. The researchers found that BIX caused death of NSCLC cells, especially those with mutations in the *EGFR* gene. Further investigation showed that BIX was effective even against drug-resistant NSCLC cells, by acting via a different metabolic pathway. BIX shows promise as an alternative therapy for lung cancer, to overcome drug resistance.

## Introduction

Lung cancer is a leading cause of cancer death worldwide and is generally classified as small cell or non-small cell lung cancer (SCLC or NSCLC)^1^. Approximately, 80–85% of lung cancers are NSCLCs. Despite significant diagnostic and therapeutic advances in recent decades, the overall 5-year survival rate of NSCLC is still only approximately 15%^2^. Conventional chemotherapeutic drugs such as *cis*-diamminedichloroplatinum (II) (cisplatin) and paclitaxel (Taxol) are generally used for lung cancer therapy^3^. However, primary or acquired resistance of NSCLCs to these conventional chemotherapeutic drugs is common, indicating an urgent need to identify new therapeutic targets to treat these devastating cancers.

Epidermal growth factor receptor (EGFR), a transmembrane glycoprotein with an extracellular epidermal growth factor binding domain and an intracellular tyrosine kinase domain, belongs to the ErbB family of receptor tyrosine kinases^4^. The interaction of EGFR with its ligand leads to its autophosphorylation via its intrinsic tyrosine/kinase activity, triggering several signal transduction pathways^5^. Constitutive or sustained activation of the downstream targets of EGFR is known to be associated with various cellular functions, including cell proliferation, motility, and cancer cell survival. Interestingly, approximately 10–35% of patients with lung adenocarcinoma harbor tumor-associated EGFR mutations^6,7^. EGFR mutations are located in exons 18–21, which encode a portion of the EGFR kinase domain and include exon 19 deletions (del19) and the L858R mutation in exon 21^8^. These mutations cause an increase in kinase activity, leading to constitutive activation of signal transduction pathways, which in turn induces cell proliferation or blocks the apoptotic response, regardless of the presence of extracellular ligands^9^.

Chemotherapeutic drugs targeting the kinase activity of EGFR, including first-generation EGFR tyrosine kinase inhibitors (TKIs) such as gefitinib and erlotinib, have been shown to be clinically successful in many NSCLC patients harboring an EGFR mutation. However, the T790M mutation leads to acquired resistance to first- and second-generation EGFR-TKIs^10–12^. Several third-generation EGFR-TKIs have recently been developed in an attempt to overcome T790M-mediated resistance, but although they proved to be effective initially, as found for first- and second-generation EGFR-TKIs, the occurrence of additional mutations such as C797S in EGFR or FGFR1 gene amplification induced acquired resistance to these new drugs^13^. Hence, there is a strong impetus to identify new therapeutic approaches to overcome acquired resistance to EGFR-TKIs in lung cancer patients.

BIX was the first small molecule G9a inhibitor to be developed^14^ and exerts antitumor effects on various cancer cells. BIX activates apoptosis via a USP9X-mediated reduction in Mcl-1 expression in bladder cancer^15^, induces autophagy-mediated cell death via accumulation of reactive oxygen species (ROS) in breast cancer^16,17^, and upregulates p53 expression in colon cancer^18^. In addition, BIX induces TRAIL-induced apoptosis via downregulation of survivin and upregulation of DR5 expression in renal cancer^19^. Of note, BIX was found to suppress cell proliferation and trigger apoptotic cell death in lung cancer by downregulating Bcl-2 expression^20^. Furthermore, BIX was shown in another report to reduce the viability of NSCLC cells through induction of autophagy^21^. However, the precise mechanisms underlying this antitumor activity of BIX, particularly in relation to EGFR signaling, remain largely unknown.

In our current study, we demonstrate for the first time that BIX has inhibitory effects on lung cancer metabolism. We found from our analyses that BIX induces apoptotic cell death only in EGFR-mutant NSCLC cells by inhibiting the activity of Jumonji histone demethylases, particularly KDM3A, rather than through blockade of the G9a histone methyltransferase. Interestingly, BIX exposure also causes a significant reduction in the BCKDHA level, leading to reduced branched-chain amino acid (BCAA) metabolism-mediated TCA cycle fueling. We further found that suppression of BCAA metabolism also dramatically inhibited EGFR signaling, which in turn induced apoptosis. Significantly, BIX overcomes acquired resistance to EGFR-TKIs and is thus a possible new therapeutic approach for EGFR-mutant NSCLC.

## Materials and methods

### Cell culture

EGFR-WT (H460 and A549) and EGFR-mutant (H1975; L858R + T790M) NSCLC cells were acquired from the American Type Culture Collection. The PC-9 (del19) cell line was a kind gift from Dr. Kazuto Nishio (National Cancer Center Hospital Tokyo, Japan) and has been previously characterized^6,22^. PC-9/GR (a gefitinib-resistant cell line) and PC-9/ER (an erlotinib-resistant cell line) cells were established as part of a previous study^23^. All cells were maintained at 37 °C in humidified air with 5% CO_2_ and grown in RPMI 1640 medium (Thermo Scientific, Waltham, MA) supplemented with 10% fetal bovine serum (FBS), 100 U/mL penicillin, and 100 μg/mL streptomycin (Thermo Scientific). Cell cultures were routinely tested for mycoplasma contamination.

### Reagents and antibodies

BIX01294 (8006) was purchased from Selleckchem (Houston, TX). UNC0642 (14604) was purchased from Cayman Chemical Company (Ann Arbor, MI), and JIB04 (4972) was obtained from Tocris Bioscience (Bristol, United Kingdom). Antibodies against AKT (9272), p-AKT (4060), PARP (9542), cleaved PARP (9541), caspase-3 (9662), cleaved caspase-3 (9661), ERK (9102), and p-JNK (4668) were purchased from Cell Signaling Technology (Beverly, MA). Antibodies against β-actin (sc-47778), EGFR (sc-03), p-EGFR (sc-12351), p-ERK (sc-7383), BCKDHA (sc-271538) and JNK (sc-7345) were obtained from Santa Cruz Biotechnology (Dallas, TX). The antibody against KDM3A (12835-1-AP) was purchased from Proteintech (Rosemont, IL), and that against EHMT2 (07-551) was obtained from Sigma–Aldrich (St. Louis, MO).

### Lentivirus-mediated shRNA delivery

The RNA Interference (RNAi) Consortium clone IDs for the shRNAs used in this study were as follows: shEHMT2-1 (TRCN0000115667), shEHMT2-2 (TRCN0000115669), shBCKDHA-1 (TRCN0000028398), shBCKDHA-2 (TRCN0000028456), shKDM3A-1 (TRCN0000329990), and shKDM3A-2 (TRCN0000329992).

### Quantitative real-time PCR

Total RNA was isolated using TRIzol reagent (QIAGEN, Hilden, Germany), and cDNA was synthesized using 2 µg aliquots of these RNA preparations and MMLV HP reverse transcriptase (Epicentre, Madison, WI). Quantitative real-time PCR was performed with SYBR Green dye using an AriaMx Real-Time PCR system (Agilent Technologies, Santa Clara, CA). The relative amounts of cDNA were determined with the comparative Ct method using 18S ribosomal RNA sequences as a control. The primer sequences for BCKDHA amplification were as follows: forward, GATTTGGAATCGGAATTGCGG; reverse, CAGAGCGATAGCGA TACTTGG.

### Metabolomic analysis

Targeted liquid chromatography–tandem mass spectrometry (LC–MS/MS) metabolomic analysis was performed as previously described^24^. Briefly, cells were grown to ~60% confluence in growth medium in 10 cm dishes. After 24 h, cells were washed several times with phosphate-buffered saline (PBS) and water, harvested using 1.4 mL of cold methanol/H_2_O (80/20, v/v), and lysed by vigorous vortexing. A 100 μL aliquot of a 5 μM internal standard was then added. Metabolites were recovered by liquid–liquid extraction from the aqueous phase after the addition of chloroform. The aqueous phase was dried using vacuum centrifugation, and the sample was reconstituted with 50 μL of 50% methanol prior to LC–MS/MS analysis. The LC–MS/MS system was equipped with an Agilent 1290 HPLC instrument (Agilent Technologies, Santa Clara, CA), QTRAP 5500 mass spectrometer (AB Sciex, Concord, Ontario, Canada), and reversed-phase column (Synergi Fusion-RP 50 × 2 mm).

### ECAR and OCR measurements

The extracellular acidification rate (ECAR) and oxygen consumption rate (OCR) were measured with an XF24 extracellular flux analyzer (Seahorse Bioscience, North Billerica, MA). Briefly, cells were plated in a 24-well Seahorse plate and cultured at 37 °C in 5% CO_2_. The medium was replaced the following day with unbuffered DMEM, and the cells were incubated at 37 °C without CO_2_ for 1 h. For OCR measurements, oligomycin, FCCP, and rotenone were added to final concentrations of 0.5 μg/ml, 1 μM, and 1 μM, respectively. For ECAR measurements, glucose, oligomycin, and 2-DG were added to final concentrations of 10 mM, 1 μM, and 20 mM, respectively.

### Apoptosis quantitation

Apoptotic cell death was detected using an Annexin-V/FITC assay. Briefly, cells were harvested by trypsinization, washed with PBS, and resuspended in annexin-V binding buffer (10 mM HEPES (pH 7.4), 140 mM NaCl, 2.5 mM CaCl_2_) containing annexin-V FITC and propidium iodide. The stained cells were then quantified and analyzed in a flow cytometer (Beckman Coulter, Brea, CA).

### Quantitation of intracellular ATP

Intracellular ATP concentrations were measured using an ATP Colorimetric/Fluorometric Assay Kit (Biovision Incorporated, Milpitas, CA) in accordance with the manufacturer’s instructions. Briefly, cells were lysed in 100 μl of ATP assay buffer, and 50 μl aliquots of the supernatants were collected and added to a 96-well plate. We then added 50 μl of ATP assay buffer containing the ATP probe, ATP converter, and developer to each well. The absorbance was then measured at 570 nm.

### Xenograft experiments

Female severe combined immunodeficiency mice were purchased from Joong Ah Bio. All experimental procedures were approved by the Institutional Animal Care and Use Committee of the Asan Institute for Life Sciences (protocol 2021-02-041, 2021-02-021). A total of 1 × 10^6^ cells suspended in 100 μL of Hank’s buffered saline solution were injected subcutaneously into the lower flank of each mouse (five mice per group). The mice in each group were treated with BIX01294 (5 mg/kg or 10 mg/kg, intraperitoneally, 2 days a week) for 4 weeks when the tumor volume reached 50–100 mm^3^. The length (*L*) and width (*W*) of each tumor were measured using calipers, and the tumor volume (TV) was calculated as TV = (*L* × W^2^)/2.

### Immunohistochemistry

Xenograft tumors were excised from the animals and washed with PBS. Tumor Section (4 µM) fixed with 4% paraformaldehyde (PFA) and embedded in paraffin were deparaffinized, rehydrated and subjected to antigen retrieval. An antibody against total EGFR (sc-373746) was obtained from Santa Cruz Biotechnology (Dallas, TX). Slides were incubated with the primary antibody, washed with PBST (0.05% Tween 20 in PBS) and incubated with a biotinylated secondary antibody (Dako, Glostrup, Denmark). Slides were developed using a DAB detection kit (DAB; Dako) and counterstained with hematoxylin.

### Chromatin immunoprecipitation (ChIP) assay

Chromatin immunoprecipitation was performed using a Maga ChIP G Kit (Merck Millipore, Burlington, MA) in accordance with the manufacturer’s directions. Briefly, cells (1 × 10^7^ cells) were treated with 1% formaldehyde and sonicated. Soluble chromatin extracts containing DNA fragments were immunoprecipitated by using an anti-H3K9me2 antibody (ab1220, Abcam), an anti-KDM3A antibody (12835-1-ap, Proteintech), and normal rabbit/mouse IgG as negative controls (sc2027, sc2025; Santa Cruz). The immunoprecipitated DNA was then analyzed by real-time PCR. Primers specific for BCKDHA were used, as follows: sense, 5′-CGTCAGGCACATAAAGAGGC-3′; antisense, 5′-CACAGATCTAGCCAGTCCCC-3′.

### Statistical analysis

Data are presented as the mean ± standard deviation values. All comparisons were performed using unpaired Student’s *t* test.

## Results

### BIX induces apoptosis only in EGFR-mutant NSCLC cells via a reduction in the EGFR level

**Fig. 1:**
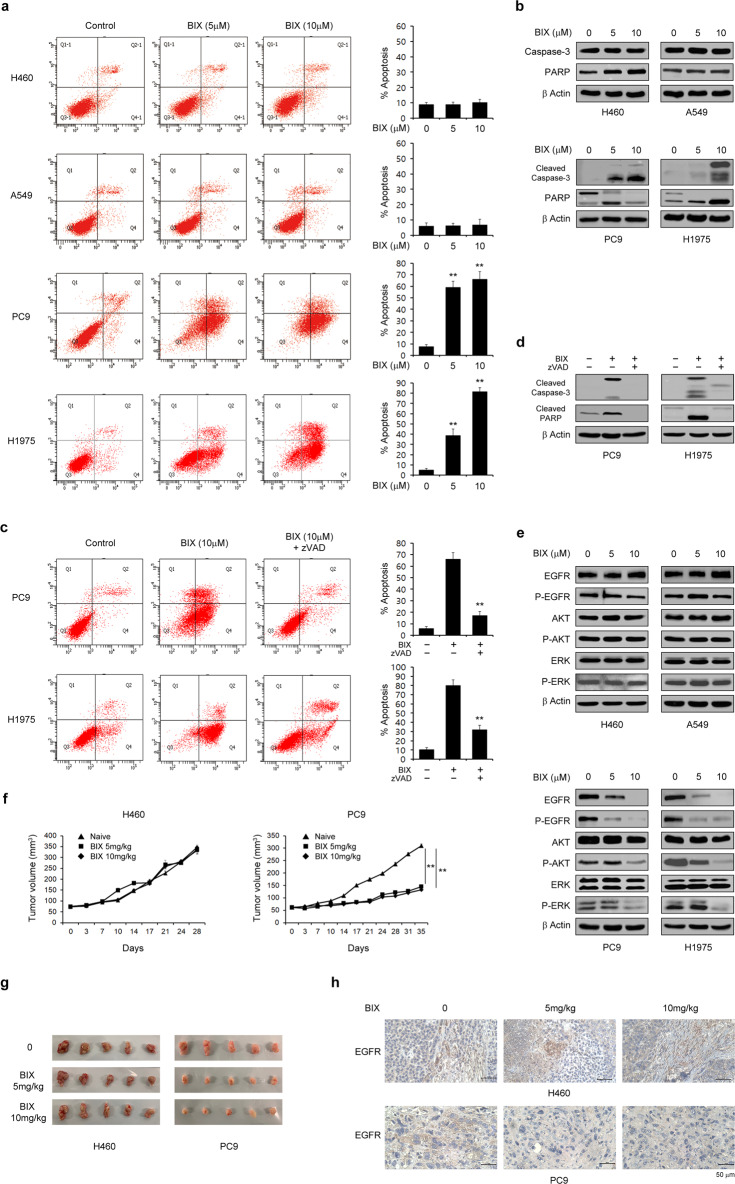
BIX selectively sensitizes EGFR-mutant NSCLC cells to apoptosis. **a** EGFR-WT and EGFR-mutant NSCLC cells were treated with the indicated concentrations of BIX for 48 h and assayed for cell death by annexin V/PI staining and flow cytometry. The error bars indicate the s.d. of triplicate wells from a representative experiment. **b** EGFR-WT and EGFR-mutant NSCLC cells were treated with the indicated concentration of BIX for 48 h and immunoblotted with the indicated antibodies. **c** EGFR-mutant NSCLC cells were treated with BIX (10 µM) for 48 h in the presence or absence of zVAD-fmk (50 μM) and assayed for cell death by annexin V/PI staining and flow cytometry. The error bars indicate the s.d. of triplicate wells from a representative experiment. **d** EGFR-mutant NSCLC cells were treated with BIX (10 µM) for 48 h in the presence or absence of zVAD-fmk (50 μM) and immunoblotted with the indicated antibodies. **e** EGFR-WT and mutant NSCLC cells were treated with the indicated concentration of BIX for 48 h and immunoblotted with the indicated antibodies. **f** SCID mice bearing established PC9 and H460 tumor xenografts were treated with BIX. Tumor volumes were calculated on the indicated days. **g** Representative images of xenograft tumors excised from the mice**. h** The expression of EGFR was determined using IHC staining in tumor tissue sections from xenografts; **p < 0.01.

To evaluate the antitumor effects of BIX on NSCLC cell survival, we first examined its effects on cell death in these cells. As shown in Fig. 1a, BIX had no significant apoptotic effect on EGFR-WT NSCLC cells (H460 and A549), whereas it caused a dramatic increase in apoptotic cell death in EGFR-mutant NSCLC cells (PC9 and H1975). Consistent with these results, treatment with BIX activated effector caspase-3 and poly (ADP-ribose) polymerase (PARP) in EGFR-mutant NSCLC cells but not in EGFR-WT NSCLC cells (Fig. 1b). Furthermore, apoptotic cell death induced by BIX was completely inhibited by treatment with benzyloxycarbonyl-Val-Ala-Asp-(OMe) fluoromethyl ketone (zVAD-fmk; Fig. 1c, d), indicating that BIX induces apoptotic cell death in EGFR-mutant NSCLC cells in a caspase-dependent manner.

EGFR-mutant NSCLCs, which depend on EGFR for survival, rely more strongly on EGFR signaling than do their WT counterparts^25–27^. Given our finding that BIX led to robust apoptotic cell death only in EGFR-mutant NSCLCs, we speculated that it may also have an inhibitory effect on EGFR signaling. Interestingly, treatment with BIX did not significantly affect EGFR signaling in EGFR-WT NSCLCs, whereas it markedly decreased the EGFR level and inhibited the phosphorylation of the EGFR signaling components AKT and ERK in EGFR-mutant NSCLC cells (Fig. 1e). To further confirm the antitumor effects of BIX on the survival of EGFR-mutant NSCLC cells, we assessed the ability of these cells to grow in vivo as xenografts. As shown in Fig. 1f, BIX treatment significantly diminished the growth of EGFR-mutant NSCLC cells (PC9) compared with EGFR-WT NSCLC cells (H460) in vivo. In addition, the size of the xenograft tumors was significantly reduced only in mice implanted with EGFR-mutant NSCLC cells following BIX treatment (Fig. 1g). Immunohistochemical (IHC) analysis showed that BIX treatment obviously reduced the expression of EGFR in EGFR-mutant NSCLC tumors compared with EGFR-WT NSCLC tumors (Fig. 1h). Taken together, these data demonstrated that BIX triggers apoptotic cell death in EGFR-mutant NSCLC cells by inhibiting EGFR signaling.

### Normal mitochondrial function is required for maintaining the EGFR level

**Fig. 2:**
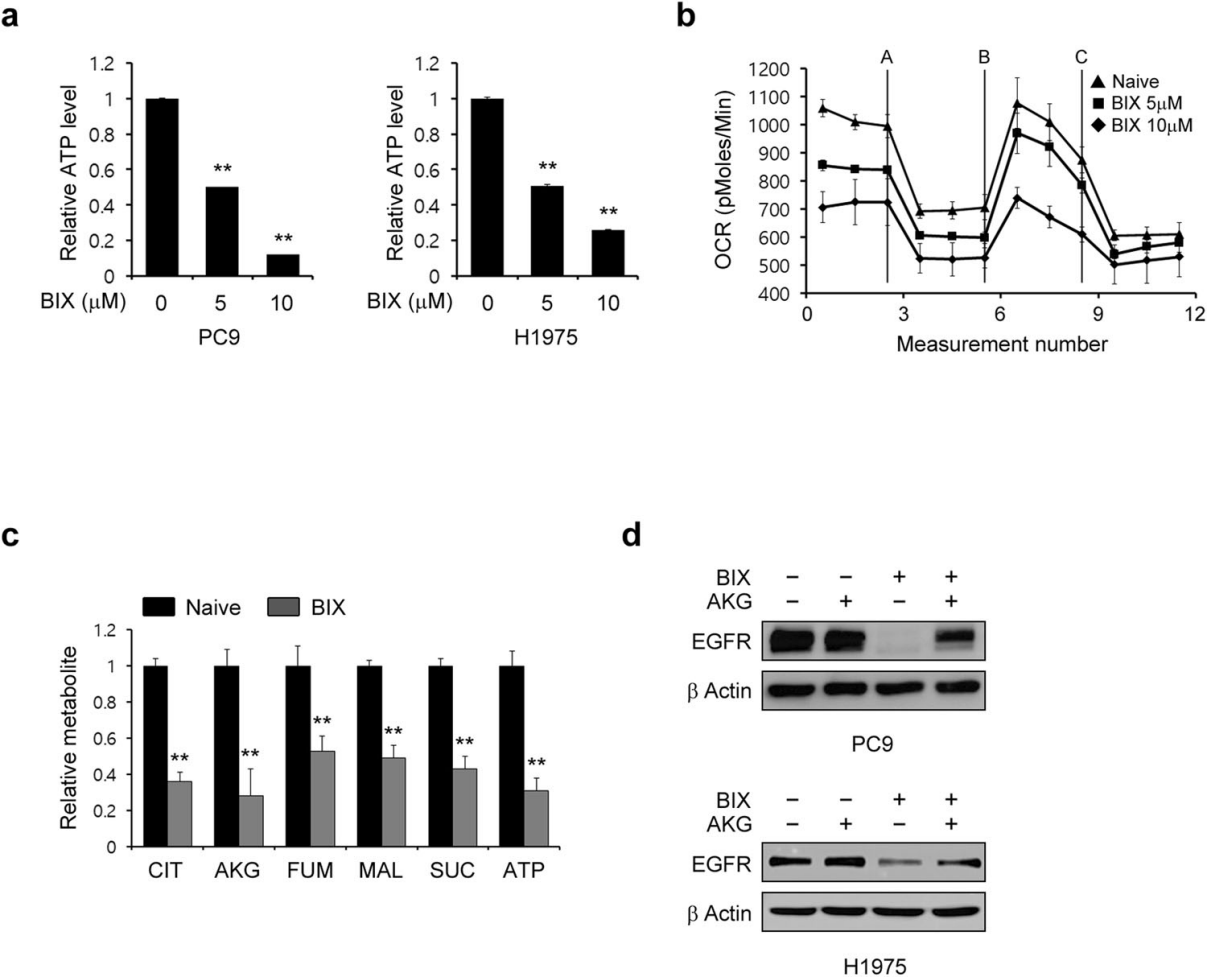
BIX suppresses normal mitochondrial function. **a** EGFR-mutant NSCLC cells were treated with the indicated concentration of BIX for 48 h and assayed for intracellular ATP. **b** PC9 cells were treated with the indicated concentration of BIX for 48 h, and the oxygen consumption rate was measured with an extracellular flux analyzer. **c** TCA cycle metabolite pools in PC9 cells treated with BIX (10 µM) were analyzed using LC–MS/MS. **d** EGFR-mutant NSCLC cells were treated with BIX (10 µM) in the presence or absence of α-ketoglutarate (AKG; 2 mM) and immunoblotted with the indicated antibodies. The error bars indicate the s.d. of triplicate wells from a representative experiment; **p < 0.01.

We previously demonstrated that inhibition of mitochondrial ATP production robustly decreases the EGFR level and subsequently induces apoptotic cell death, indicating that mitochondrial ATP production is critical for maintaining the EGFR level^24^. In our current experiments, we thus tested whether BIX affects mitochondrial function. As shown in Fig. 2a, the ATP level was significantly reduced in the presence of BIX. Oxygen consumption was also found to be markedly decreased upon BIX treatment (Fig. 2b). We next investigated the impact of BIX on mitochondrial metabolism using LC–MS/MS metabolomics analysis to explore its direct effects on mitochondrial ATP production. BIX treatment led to significant reductions in the levels of the tricarboxylic acid cycle (TCA) cycle intermediates (Fig. 2c). To test whether the BIX-mediated reduction in the EGFR level as a result of insufficient maintenance of mitochondrial ATP production, we attempted to reverse this reduction with α-ketoglutarate supplementation and indeed observed significant restoration of the EGFR level, which was decreased following BIX exposure (Fig. 2d). Hence, our results suggested that BIX inhibits mitochondrial metabolism, which in turn suppresses EGFR signaling.

### BIX inhibits the supply of a carbon source for the TCA cycle through suppression of BCAA metabolism

**Fig. 3:**
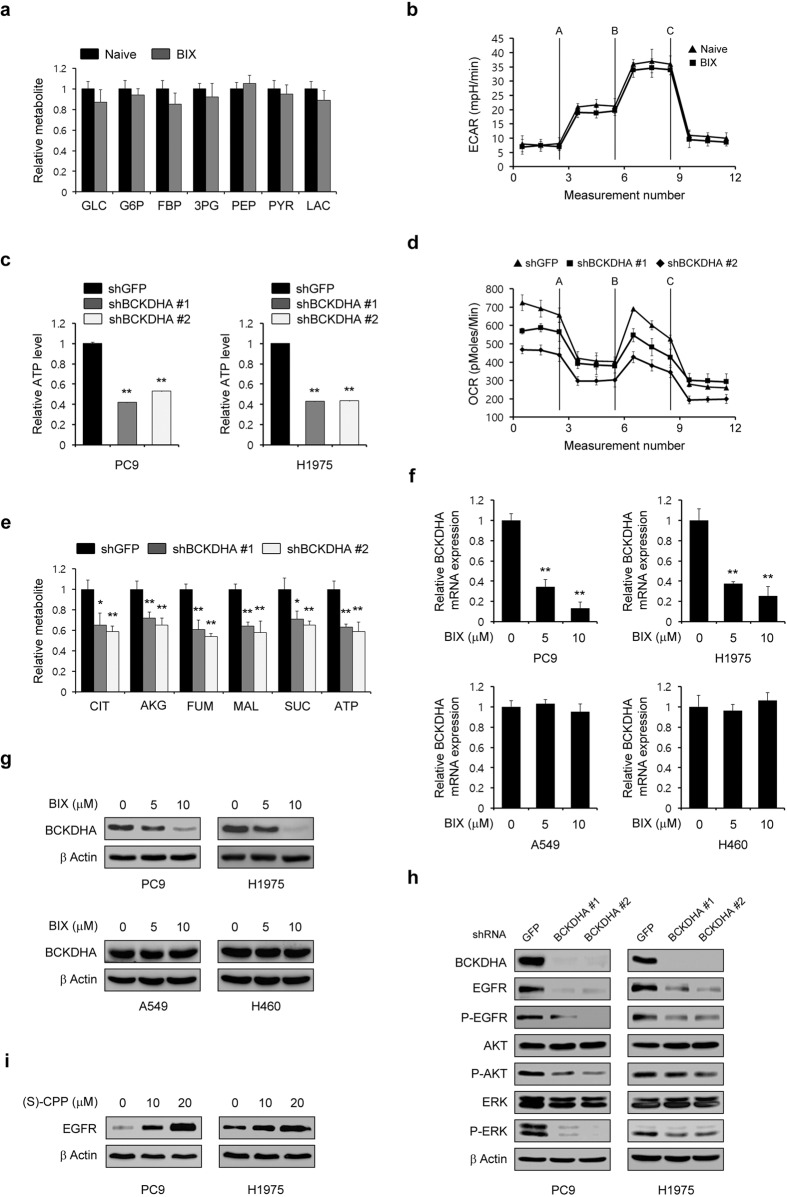
Maintenance of the EGFR level requires BCAA metabolism as a carbon source for the TCA cycle. **a** Glycolytic metabolite pools in PC9 cells treated with BIX (10 µM) were analyzed via LC–MS/MS. **b** PC9 cells were treated with BIX (10 µM) for 48 h, and the real-time glycolytic rate was determined using an extracellular flux analyzer. **c** EGFR-mutant NSCLC cells expressing control (shGFP) or BCKDHA shRNAs (shBCKDHAs) were assayed for intracellular ATP. **d** The oxygen consumption rate in PC9 cells expressing control (shGFP) or BCKDHA shRNAs (shBCKDHAs) was measured with an extracellular flux analyzer. **e** TCA metabolite pools in PC9 cells expressing control (shGFP) or BCKDHA shRNAs (shBCKDHAs) were analyzed via LC–MS/MS. **f** EGFR-WT and mutant NSCLC cells were treated with the indicated concentration of BIX for 48 h, and the expression of BCKDHA was determined by quantitative RT–PCR. **g** EGFR-WT and mutant NSCLC cells were treated with the indicated concentration of BIX for 48 h and immunoblotted with an anti-BCKDHA antibody. **h** EGFR-mutant NSCLC cells expressing control (shGFP) or BCKDHA shRNAs (shBCKDHAs) were immunoblotted with the indicated antibodies. **i** EGFR-mutant NSCLC cells were treated with the indicated concentration of (S)-CCP for 48 h and immunoblotted with an anti-EGFR antibody. The error bars indicate the s.d. of triplicate wells from a representative experiment; *p < 0.05, **p < 0.01.

Considering the findings of a previous study indicating that enhanced glycolysis is indispensable for maintaining the EGFR level in EGFR-mutant NSCLC cells by fueling the TCA cycle^24^, we next tested whether BIX inhibits glucose metabolism. As shown in Fig. 3a, the glycolytic metabolite levels were unchanged upon BIX treatment. Consistent with this result, BIX had no significant effect on the extracellular acidification rate (Fig. 3b), suggesting that it has no impact on glucose metabolism. Given that BCAAs fuel the TCA cycle through the transfer of respective amino groups^28^, we next examined the ATP level upon the knockdown of branched-chain α-keto acid dehydrogenase a (BCKDHA), the enzymatic catalyst for the second step of the BCAA catabolic pathway. This was done to investigate the functional role of BCKDHA in TCA metabolism. We found that BCKDHA knockdown led to a significant decrease in the ATP level (Fig. 3c). The oxygen consumption rate also decreased upon BCKDHA knockdown (Fig. 3d). In addition, the levels of TCA cycle intermediates were significantly changed upon BCKDHA knockdown (Fig. 3e), suggesting that BCAA metabolism is an important source of carbon for the TCA cycle. We further found that BIX reduced BCKDHA mRNA and protein levels only in EGFR-mutant NSCLC cells, indicating that this small molecule inhibitor affects BCAA metabolism in these NSCLC cells (Fig. 3f, g). In addition, BCKDHA knockdown dramatically decreased the EGFR level and inhibited AKT and ERK phosphorylation (Fig. 3h). Furthermore, the EGFR level was significantly increased in a dose-dependent manner upon treatment with (S)-CPP, an inhibitor of the branched-chain α-ketoacid dehydrogenase complex (BCKDC) kinase that blocks BCKDH activity (Fig. 3i). These data collectively demonstrated that BCAA metabolism, as a source of fuel for the TCA cycle, is critical for maintaining the EGFR level.

### BIX decreases the BCKDHA level by inhibiting Jumonji histone demethylase activity but not G9α activity

**Fig. 4:**
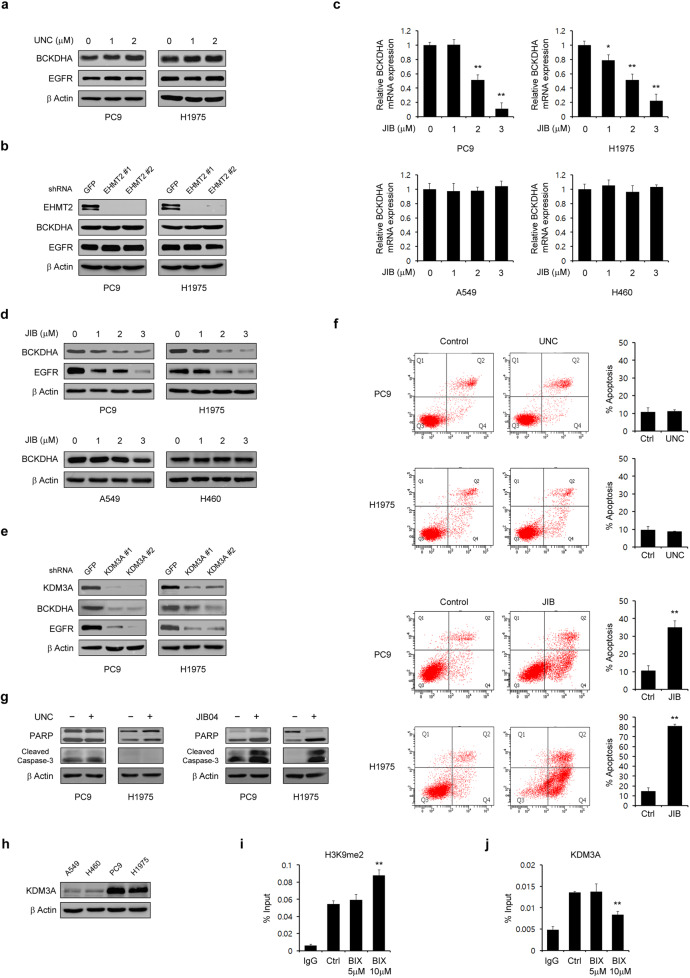
BIX regulates the BCKDHA level through inhibition of Jumonji demethylase activity. **a** EGFR-mutant NSCLC cells were treated with the indicated concentration of UNC for 48 h and immunoblotted with the indicated antibodies. **b** EGFR-mutant NSCLC cells expressing control (shGFP) or EHMT2 shRNAs (shEHMT2s) were immunoblotted with the indicated antibodies. **c** The expression of BCKDHA was determined by quantitative RT–PCR in EGFR-WT and mutant NSCLC cells treated with the indicated concentration of JIB. **d** EGFR-WT and mutant NSCLC cells were treated with the indicated concentration of JIB for 48 h and immunoblotted with the indicated antibodies. **e** EGFR-mutant NSCLC cells expressing control (shGFP) or KDM3A shRNAs (shKDM3As) were immunoblotted with the indicated antibodies. **f** EGFR-mutant NSCLC cells were treated with either UNC (2 µM) or JIB (3 µM) for 48 h and assayed for cell death by annexin V/PI staining and flow cytometry. **g** EGFR-mutant NSCLC cells were treated with either UNC (2 µM) or JIB (3 µM) for 48 h and immunoblotted with the indicated antibodies. **h** Western blot analysis of KDM3A expression in EGFR-WT and mutant NSCLC cells. **i**, **j** PC9 cells treated with BIX were subjected to ChIP with anti-H3K9me2 or anti-KDM3A antibodies. KDM3A gene expression was analyzed by quantitative RT–PCR. The error bars indicate the s.d. of triplicate wells from a representative experiment; *p < 0.05; **p < 0.01. UNC, UNC0642; JIB, JIB04.

We next explored the mechanisms by which BIX regulates the BCKDHA level. We first tested whether another selective inhibitor of the G9a histone methyltransferase, UNC0624, can reduce the BCKDHA level, because BIX is a well-known G9a inhibitor. Unexpectedly, both the BCKDHA and EGFR levels were unchanged by UNC0624 treatment (Fig. 4a). Consistent with this result, knockdown of the G9a histone methyltransferase, i.e., EHMT2, had no effect on the level of either BCKDHA or EGFR (Fig. 4b), indicating that BIX does not downregulate BCKDHA through its G9a histone methyltransferase inhibitory function.

It has been reported that BIX selectively inhibits a family of histone H3 lysine 9 Jumonji demethylases^29^, and we thus tested whether JIB04, a pan-selective Jumonji histone demethylase inhibitor, downregulates BCKDHA. As shown in Fig. 4c, the BCKDHA mRNA level was significantly reduced in a dose-dependent manner upon JIB04 treatment only in EGFR-mutant NSCLC cells. Consistent with this result, JIB04 treatment reduced both the BCKDHA and EGFR protein levels in these NSCLC cells but did not reduce the protein level of BCKDHA in EGFR-WT NSCLC cells (Fig. 4d). Furthermore, knockdown of one of the Jumonji demethylases, KDM3A (lysine demethylase 3A), led to robust decreases in both the BCKDHA and EGFR levels (Fig. 4e). Given that JIB04 but not UNC0624 reduced both the BCKDHA and EGFR levels, we speculated that only JIB04 induces apoptotic cell death in EGFR-mutant NSCLC cells. Indeed, treatment with UNC0624 had no significant apoptotic effect on EGFR-mutant NSCLC cells, whereas JIB04 treatment resulted in a dramatic increase in apoptotic cell death in EGFR-mutant NSCLC cells (Fig. 4f). Moreover, PARP and caspase-3 were cleaved only upon JIB04 treatment (Fig. 4g). Taken together, these data demonstrated that BIX triggers apoptotic cell death by inhibiting Jumonji demethylase activity.

For further confirmation of the KDM3A-mediated reduction in BCKDHA expression, we first compared the expression level of KDM3A between EGFR-mutant and EGFR-WT NSCLC cells. Compared with EGFR-WT NSCLC cells, EGFR-mutant NSCLC cells exhibited increased expression of KDM3A (Fig. 4h). We next performed chromatin immunoprecipitation (ChIP) assay to assess histone H3 modification in the BCKDHA promoter and the binding of KDM3A to the BCKDHA gene promoter. As shown in Fig. 4i, j, H3K9me2 was elevated upon BIX treatment, and BIX treatment inhibited KDM3A binding to the BCKDHA promoter. Therefore, these results indicated that the BIX-mediated BCKDHA reduction results from its inhibition of KDM3A.

### BIX overcomes acquired resistance to EGFR-TKIs

**Fig. 5:**
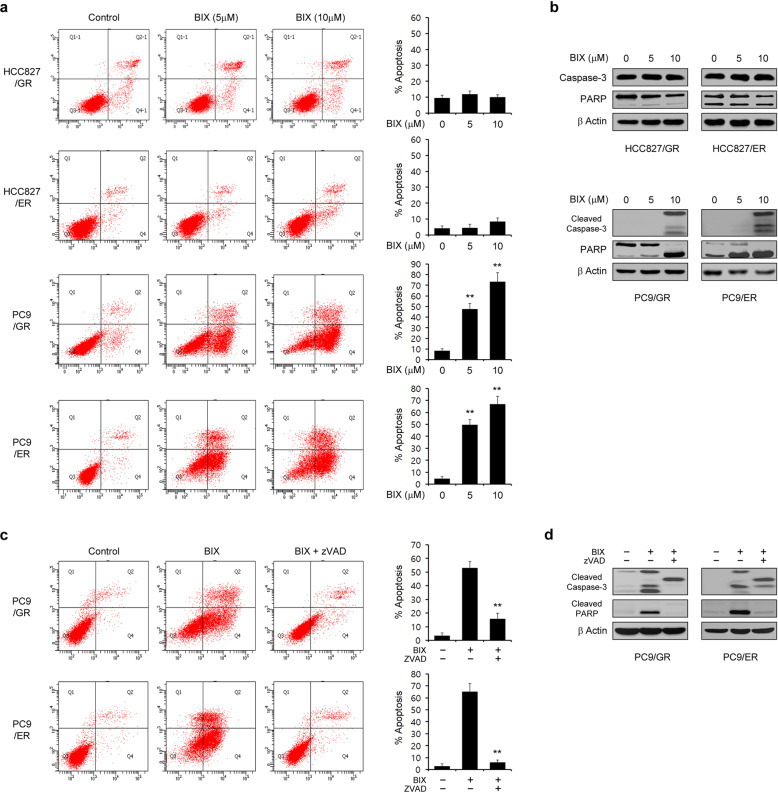
BIX sensitizes EGFR-TKI-resistant NSCLC cells to apoptosis. **a** EGFR-TKI-resistant NSCLC cells were treated with the indicated concentration of BIX for 48 h and assayed for cell death by annexin V/PI staining and flow cytometry. **b** EGFR-TKI-resistant NSCLC cells were treated with the indicated concentration of BIX for 48 h and immunoblotted with the indicated antibodies. **c** EGFR-TKI-resistant NSCLC cells were treated with BIX (10 µM) for 48 h in the presence or absence of zVAD-fmk (50 μM) and assayed for cell death by annexin V/PI staining and flow cytometry. **d** EGFR-TKI-resistant NSCLC cells were treated with BIX (10 µM) for 48 h in the presence or absence of zVAD-fmk (50 μM) and immunoblotted with the indicated antibodies. The error bars indicate the s.d. of triplicate wells from a representative experiment; **p < 0.01. HCC827/GR, HCC827/gefitinib-resistant; HCC827/ER, HCC827/erlotinib-resistant; PC9/GR, PC9/gefitinib-resistant; PC9/ER, PC9/erlotinib-resistant.

We previously reported that EGFR knockdown had no significant effect on the proliferation of cells with MET- or AXL-mediated EGFR-TKI resistance (HCC827/GR and HCC827/ER cells), whereas it markedly inhibited the proliferation of cells with T790M-mediated EGFR-TKI resistance (PC-9/GR and PC-9/ER cells)^24^. Because BIX reduces the EGFR level and inhibits EGFR signaling, we suspected that it may affect PC-9/GR and PC-9/ER cell survival. As shown in Fig. 5a, BIX treatment did not induce apoptotic cell death in HCC827/GR and HCC827/ER cells but did so in PC-9/GR and PC-9/ER cells. Consistent with these results, PARP and caspase-3 were cleaved only in PC-9/GR and PC-9/ER cells upon BIX treatment (Fig. 5b). We next tested whether the cell death caused by BIX was caspase-dependent. Treatment with zVAD-fmk significantly inhibited the apoptotic response (Fig. 5c) and the cleavage of PARP and caspase-3 (Fig. 5d) induced by BIX. These data thus demonstrated that BIX treatment can overcome acquired resistance to EGFR-TKIs.

### BIX induces apoptosis in EGFR-TKI-resistant NSCLC cells by inhibiting epigenetic regulation of BCKDHA expression

**Fig. 6:**
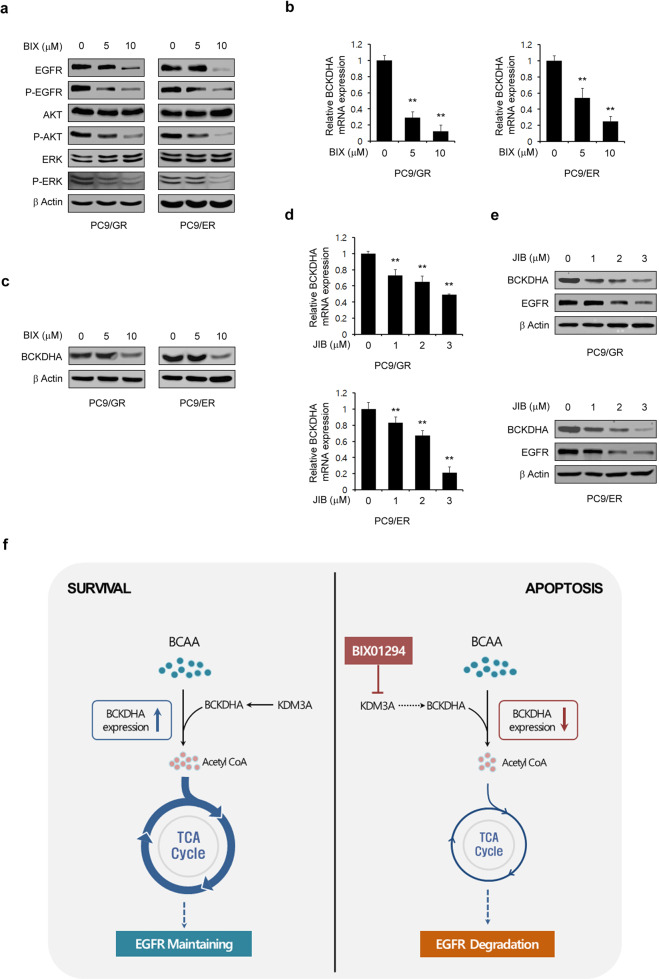
BIX reduces the BCKDHA level in EGFR-TKI-resistant NSCLC cells that are dependent on EGFR signaling. **a** EGFR-TKI-resistant NSCLC cells were treated with the indicated concentration of BIX for 48 h and immunoblotted with the indicated antibodies. **b** The expression of BCKDHA was determined by quantitative RT-PCR in EGFR-TKI-resistant NSCLC cells treated with the indicated concentration of BIX. **c** EGFR-TKI-resistant NSCLC cells were treated with the indicated concentration of BIX for 48 h and immunoblotted with the indicated antibodies. **d** The expression of BCKDHA was determined by quantitative RT-PCR in EGFR-TKI-resistant NSCLC cells treated with the indicated concentration of JIB04. **e** EGFR-TKI-resistant NSCLC cells were treated with the indicated concentration of JIB for 48 h and immunoblotted with the indicated antibodies. **f** Model of BIX-mediated apoptosis activation in EGFR-mutant NSCLC cells through a BCKDHA-mediated reduction in the EGFR level. The error bars indicate the s.d. of triplicate wells from a representative experiment; **p < 0.01.

We next investigated the mechanisms by which BIX overcomes acquired resistance to EGFR-TKIs. Consistent with our initial observation (Fig. 1e), treatment with BIX markedly decreased the EGFR level in a dose-dependent manner and inhibited the phosphorylation of the EGFR signaling components AKT and ERK in PC9/GR and PC9/ER cells (Fig. 6a). We then explored the involvement of BCKDHA in BIX-mediated apoptotic cell death in PC-9/GR and PC-9/ER cells. Treatment with BIX reduced the BCKDHA transcript level in a dose-dependent manner in PC9/GR and PC9/ER cells (Fig. 6b). BIX treatment also led to dose-dependent decreases in the protein levels of BCKDHA and EGFR (Fig. 6c). Furthermore, and consistent with our prior observations (Fig. 4c, d), we found that inhibition of Jumonji histone demethylase activity with JIB04 resulted in a decrease in the mRNA level of BCKDHA (Fig. 6d) and reduced the protein levels of BCKDHA and EGFR in a dose-dependent manner in PC9/GR and PC9/ER cells (Fig. 6e). Hence, it appears that BIX overcomes acquired resistance to EGFR-TKIs via a Jumonji demethylase-mediated reduction in the EGFR level.

Collectively, the findings of our study showed that BIX induces apoptotic cell death in EGFR-mutant NSCLC cells but not in their wild-type counterparts. We also found that BIX overcomes acquired resistance to EGFR-TKIs. BIX reduced the BCKDHA level by inhibiting Jumonji histone demethylase activity, leading to a reduction in the supply of a carbon source for the TCA cycle through suppression of BCAA metabolism. Inhibition of BCAA-derived mitochondrial ATP production led to a decrease in the EGFR level, which in turn induced apoptosis. Thus, BIX or Jumonji histone demethylase-mediated regulation of BCAA metabolism may provide effective future strategies for EGFR-mutant NSCLC therapy and for overcoming EGFR-TKI resistance in NSCLC patients (Fig. 6f).

## Discussion

BIX01294 has been reported to exert antitumor effects against a variety of cancers^15–19^, including lung cancer^20,21,30^, but the precise mechanisms of these effects, particularly in NSCLCs, remain unclear. In our present study, we proposed a unique mechanism by which BIX01294 has antitumor effects, particularly on EGFR-mutant NSCLCs, by blocking BCAA metabolism-mediated maintenance of the EGFR level through inhibition of the activity of Jumonji histone demethylases, particularly KDM3A.

BIX01294 is a specific inhibitor of the G9a histone methyltransferase^14^, which plays important role in DNA replication, damage and repair, and in gene expression by regulating DNA methylation^31^. Moreover, given that G9a has been shown to be overexpressed in many tumor cells and is associated with the occurrence and development of tumors, it has become a promising antitumor target^32^, and many small molecule inhibitors of G9a have been developed for evaluation as cancer therapeutics. BIX01294 was also developed as a small molecule G9a inhibitor, and most studies of this agent have reported that its antitumor effects are mediated through this function. However, BIX01294 has also been shown to selectively inhibit Jumonji histone demethylase activity^29^. Importantly, we found in our present study that the BIX01294-mediated reduction in the BCKDHA level is due to its inhibition of Jumonji histone demethylases and not its suppression of G9a (Fig. 4). Similar to G9a, a number of Jumonji C family members have been found to be overexpressed in many types of cancer^33^. Of note, the Jumonji histone demethylase KDM3A is highly expressed in breast, prostate, colon, kidney, and liver cancers^34^. KDM3A is also overexpressed in NSCLC, as it is essential for NSCLC growth^35^. In addition, a recent study reported that KDM3A regulates the expression of EGFR through Kruppel-like factor 5 and SMAD family member 4^36^. Consistent with these earlier data, we observed here that both JIB04 treatment and KDM3A knockdown led to a significant reduction in the EGFR level and to inhibition of EGFR signaling. Thus, our data and those from other studies suggest that BIX01294-mediated KDM3A inhibition may serve as an attractive therapeutic target for EGFR-mutant NSCLCs.

We previously reported that EGFR mutation-mediated upregulation of glycolysis is required for maintaining the level of EGFR as a TCA cycle fuel and that inhibition of glucose-derived mitochondrial ATP production leads to a significant decrease in the EGFR level, which in turn results in the activation of apoptosis, suggesting that the maintenance of proper mitochondrial function is critical for the survival of EGFR-mutant NSCLCs by sustaining EGFR stability^24^. We also observed that BIX01294 treatment impairs mitochondrial metabolism (Fig. 2). Based on our previous study, here, we tested whether the inhibition of mitochondrial metabolism by BIX01294 is due to blockade of glucose metabolism, but we found that BIX01294 has no significant effect on glucose metabolism (Fig. 3a, b). Cancer cells utilize a variety of carbon sources, including glucose, glutamine, other amino acids, and fatty acids, to replenish the TCA cycle^37^. Indeed, NSCLC tumors display enhanced uptake of BCAAs, which provide substrates for the TCA cycle, and inhibition of BCAA catabolism significantly suppresses NSCLC tumor growth^38^. In our current study, we observed that the BIX01294-mediated reduction in BCKDHA expression impaired mitochondrial metabolism and resulted in a significant decrease in the EGFR level, which induces apoptotic cell death (Figs. 3 and 4). This indicates that BIX01294 causes downregulation of EGFR by decreasing the BCAA-mediated fuel source for the TCA cycle. In addition, we found in our present study that BIX01294 markedly decreased the EGFR level in EGFR-mutant NSCLCs but not in EGFR-WT NSCLCs. We believe that this selectivity arises because EGFR-mutant NSCLCs have higher basal levels of KDM3A than EGFR-WT NSCLCs (Fig. 4h). For this reason, we think that BIX01294-mediated KDM3A inhibition leads to a significant decrease in both the BCKDHA and EGFR levels only in EGFR-mutant NSCLCs.

EGFR-mutant NSCLCs, which depend on EGFR for growth and survival, rely more strongly on EGFR signaling than their wild-type counterparts^25,26^. Given the importance of EGFR signaling for cell growth and survival, EGFR-TKIs have been developed and proven to be effective in patients diagnosed with EGFR-mutant NSCLC. However, acquired EGFR-TKI resistance is typical in these cases^39^. Although second- and third-generation EGFR-TKIs have been developed to try to overcome this acquired drug resistance, acquired resistance to these additional agents develops in most patients. Our current study provides reliable evidence that BIX01294 treatment can overcome T790M-mediated resistance and that EGFR stability requires the maintenance of proper mitochondrial function via BCAA-mediated fueling of the TCA cycle.
